# Isometric Mid-Thigh Pull Performance in Rugby Players: A Systematic Literature Review

**DOI:** 10.3390/jfmk5040091

**Published:** 2020-12-08

**Authors:** Eric A. Martin, George K. Beckham

**Affiliations:** Kinesiology Department, California State University, Monterey Bay, Seaside, CA 93955, USA; gbeckham@csumb.edu

**Keywords:** maximal strength, isometric strength, peak power, rate of force production, force development, rugby union, rugby league

## Abstract

The isometric mid-thigh pull (IMTP) is a multi-joint test of whole-body force production relevant to rugby players. “Rugby AND (mid-thigh pull OR midthigh pull OR mid thigh pull” were searched in PubMed, Sportdiscus, Academic Search Premier, CINAHL Plus with Full Text, and Google Scholar; the final date of search was 24 January 2018. Data extraction from 24 articles included subject characteristics, force data, and IMTP testing procedures. Select ranges of peak forces reported were: Youth: 1162–2374 N; Academy: 1855–3104 N; Professional: 2254–3851 N. Rate of force development (RFD) at 100 and 200 ms ranged from 5521 to 11,892 N and 5403 to 8405 N, respectively, among professional rugby players. Studies’ research design were of moderate quality, but most studies lacked detailed reporting of IMTP procedures. Variability of force characteristics derived from the IMTP within similar populations (e.g., approximately 200% difference in peak force between samples of professional rugby league players) as well as large and unexpected overlaps between dissimilar populations, limit conclusions about force production capabilities relative to playing level, likely due to limitations and lack of standardization of IMTP procedures. Greater uniformity in IMTP testing procedures and reporting is needed. This manuscript provides a guide for reporting needs when presenting results from an IMTP in research.

## 1. Introduction

Rugby is a collision sport played at youth, amateur, and professional levels [1,2,3,4,5,6]. There are multiple codes of rugby, including union, league, and 7s, with their own laws and strategies, but which share many similar characteristics in gameplay, technical skill, and physiological demands of players [1,2,3,4,5,6,7]. Due to the high-contact nature of rugby, the ability to generate high amounts of force in a short period of time is of substantial interest to coaches and players. Muscular strength and power have been documented as important for contact situations like tackling and scrummaging [8,9,10,11,12,13]. In these studies, strength has typically been assessed for the lower body using tests such as the barbell back squat one repetition maximum (1RM), and power has typically been assessed through some version of a vertical jump test. While both of these tests are widely used and validated [14], they only measure their one respective construct and only account for lower-body force production capabilities. While lower-body strength seems to be the most important fitness characteristic for determining tackle success, upper-body strength also plays an important role in making and breaking tackles [12]. Thus, a test of whole-body strength may be more specific to contact ability, especially for the tackle, and could be a more time-efficient option for coaches and athletes.

The isometric mid-thigh pull (IMTP) is a multi-joint exercise test to assess whole-body strength and force production capabilities. The IMTP test can be used to determine peak force (PF) development, rate of force development (RFD), and other time-constrained strength variables (e.g., force at 100 ms after the initiation of the pull) [15]. This information cannot be easily gained through standard forms of muscular strength testing, such as the barbell back squat 1RM. Other advantages to the IMTP test include time efficiency, less interruption to training, and a need for less technical instruction of the lift compared to full range of motion core lifts like squats or any of the Olympic lifts [15,16]. Drake and colleagues [16] performed a systematic literature review in an attempt to robustly examine the validity and responsiveness of isometric strength tests, including the IMTP. Of the studies Drake et al. [16] reviewed, the one with the highest methodological quality reported intraclass correlation coefficients (ICC) for inter-trial reliability at α = 0.79–0.99 for different outcomes taken from the IMTP test results of male and female National Collegiate Athletic Association Division 1 college athletes [17], indicating high reliability. Additionally, the reviewed study with the highest methodological quality that examined construct validity in rugby found a significant difference between positional groups for peak force normalized to body mass (*p* = 0.005), specifically indicating that the inside backs were stronger relative to their body mass than the tight five forwards [18]. While Drake and colleagues [16] concluded that the IMTP demonstrated moderate evidence for reliability and construct validity and recommended its use, they highlighted that the majority of studies did a poor job in reporting their methodology. One potential explanation could be a lack of widely known information on what items to report when using the IMTP test in research, thus creating a gap in knowledge among researchers and practitioners about the details of using this test.

Use of the IMTP has grown in popularity across all sports, but is increasingly used in rugby for both the reasons outlined previously, and the understanding that strength and the ability to apply forces rapidly (i.e., RFD) is of critical importance for the sport of rugby, given the number of skills for which these qualities are of value (e.g., scrum, maul, tackle). These studies have used the IMTP across a variety of rugby populations [6,18,19]. To date, there has not been any paper to summarize force production of rugby players as assessed with the IMTP test. Normative data on IMTP performance among rugby players could be useful for comparative purposes for athletes and coaches seeking to elevate their team’s performance and/or prepare their players for greater levels of play. Such a compilation would provide a benchmark of comparison for players who wish to develop their abilities needed at greater levels of play.

Recently, Comfort and colleagues [15] published an article in which they provided a summary of the methods used in IMTP research, then provided recommendations for use of the test in the future. However, in the paper they did not seek to provide any tool with which to evaluate existing literature against their recommendations; nor was there a clear recommendation of what information, at minimum, should be reported in an IMTP study. This makes it more difficult to compare results between studies and to create useful normative databases. For example, there is variety between papers regarding the inclusion or exclusion of body mass in force calculations; without explicit reporting of whether data are gross or net force, the reader could easily under or overestimate the magnitude of a given force quality that their player may need. In addition, there is substantial variation in the methods used to estimate athletes’ ability to generate force rapidly; previous studies have used peak rate of force development (highest rate of change in force over 5 ms/10 ms) or average rate of force development (e.g., average rate of change in force from onset of pull to a predetermined time point) [20,21]. These different specific measures have sometimes been conflated and described under the umbrella term “rate of force development;” although each represent slightly different aspects of rapid force generation capacity and have significant variability in inter-trial reliability [21], so clear reporting is critical for helping readers identify when different types of early force-time measures are used.

The purposes of this study were twofold: (1) to summarize any data collected using the IMTP test in any rugby players, with the goal of creating normative data for this test for coaches and players to use, and (2) to critique the methodology studies used and reported in conducting their research.

## 2. Materials and Methods

**Figure 1:**
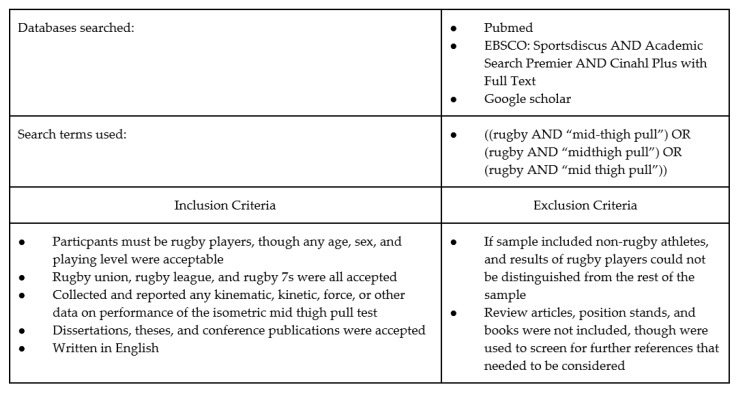
Search Strategy.

**Figure 2:**
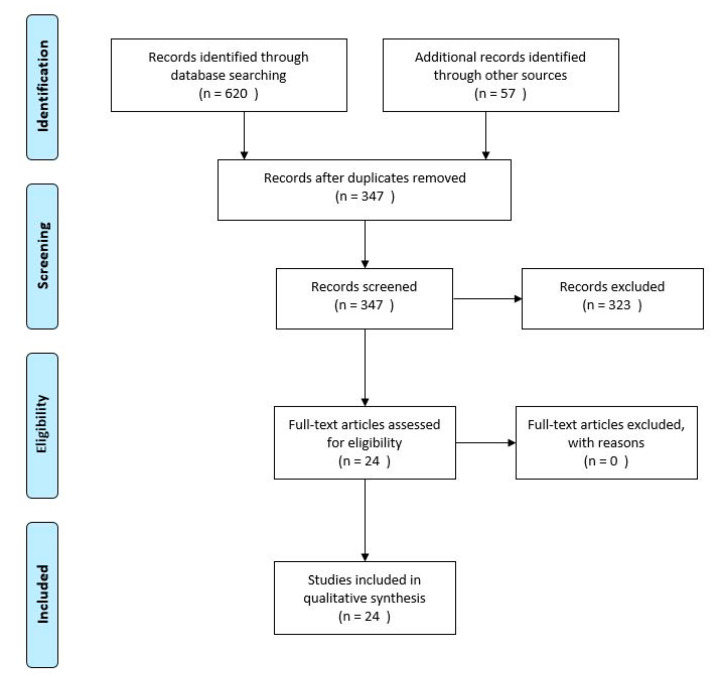
PRISMA Screening Flow Diagram. PRISMA: Preferred Reporting Items for Systematic Reviews and Meta-Analysis.

The Preferred Reporting Items for Systematic Reviews and Meta-Analysis (PRISMA) guidelines were followed in performing this search and synthesis [22]. The protocol for this review was registered with PROSPERO (CRD42018089371). Searches were executed per the search strategy contained in Figure 1. The final date of search was 24 January 2018. All results returned were copied into a shared spreadsheet. The spreadsheet included a code to indicate which search was being done (database and terms used), the title of every article returned in that search, and a hyperlink to each article returned for further review. Once all results were input into the spreadsheet, the sheet was sorted by article title and duplicates removed. Three copies of the list of all articles were made to allow each investigator to independently screen all titles and abstracts for inclusion based on the criteria of interest (Figure 1). Articles considered for exclusion were highlighted to indicate potential removal. Initial screening selections between EAM and GKB were compared for discrepancies and resolved by consensus. The full-text articles of all remaining studies were retrieved and screened one last time for inclusion by all researchers simultaneously. Bibliographies of review articles identified at any time during the search and of the full-text articles included for extraction were read to identify any further articles that may need to be included. The final flow of articles included can be seen in Figure 2.

Primary outcomes for data extraction included peak force, rate of force production, instantaneous force, and impulse measures. Secondary outcomes included use of straps to secure participants to the bar, knee and hip angles during test, level of competition of sample, and timing during competitive season. In studies that reported data from assessments at multiple time points, only the baseline data were extracted.

Studies were assessed for quality and risk of bias following the Quality Assessment Tool for Observational Cohort and Cross-Sectional Studies (QAT) [23]. Based on the purpose of this study, some of the items from this tool were deemed not applicable and therefore removed. The items were:

- Question 3: Was the participation rate of eligible persons at least 50%?
  ○ This was removed as all studies used samples of convenience.
- Question 6: For the analyses in this paper, were the exposure(s) of interest measured prior to the outcome(s) being measured?
  ○ This was removed as no exposures were anticipated or sought; in the data extraction process, other questions related to exposures were operationalized as one study reporting on players of different levels (e.g., U16 and U18).
- Question 7: Was the timeframe sufficient so that one could reasonably expect to see an association between exposure and outcome if it existed?
- Question 10: Was the exposure(s) assessed more than once over time?
- Question 13: Was loss to follow-up after baseline 20% or less?
  ○ Questions 7, 10, and 13 were removed because data were extracted only for single baseline measurements.

Furthermore, two other questions about exposures were not applicable to all studies because they had a single sample level. Thus, a general methodological score out of either seven or nine points was created, and a percentage rating calculated to describe the general methodological and reporting quality of each study.

Additionally, secondary outcomes were used to assess quality of reporting of the IMTP testing procedures. The items assessed and how they were scored are detailed in Table 1. The assessment criteria were initially developed by the two principle investigators on this study and based on the recent recommendations for IMTP procedures by Comfort and colleagues [15]. The first draft of the assessment criteria was then sent to three experts in the field of strength and conditioning with extensive experience with the IMTP test (MHS, GGH, PC), who provided constructive criticisms that were incorporated into the final version of assessment criteria, thus determining face validity of the tool. Ratings for included studies are outlined in Table 2.

## 3. Results

The search strategy identified 24 research publications: 20 peer-reviewed journal articles, three dissertations/theses, and one conference poster. Two pairs of journal articles reported data on the same sample [18,25], but because they reported different outcomes, all studies were kept and used for data extraction. Eleven studies were in professional- or Olympic-level rugby players (or a mix of professional and academy players from the same club), two studies in exclusively academy players, four in university players, four in amateur players, and three in adolescent players. Over half the studies (*n* = 13) were conducted on players in the United Kingdom; four studies were conducted in the United States of America, three in New Zealand, and one each in Australia, France, and Portugal (Table 3).

**Figure 3:**
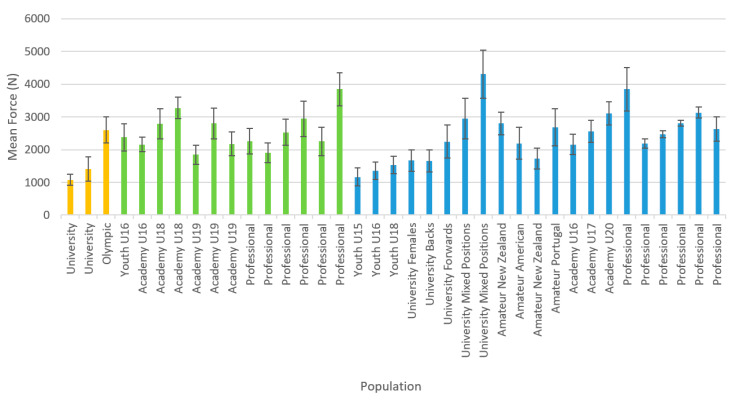
Mean Peak Total-Body Force Production of Rugby Players by Code and Playing Level. Notes: Error bars represent reported standard deviation; Orange = Rugby 7s; Green = Rugby League; Blue = Rugby Union.

**Figure 4:**
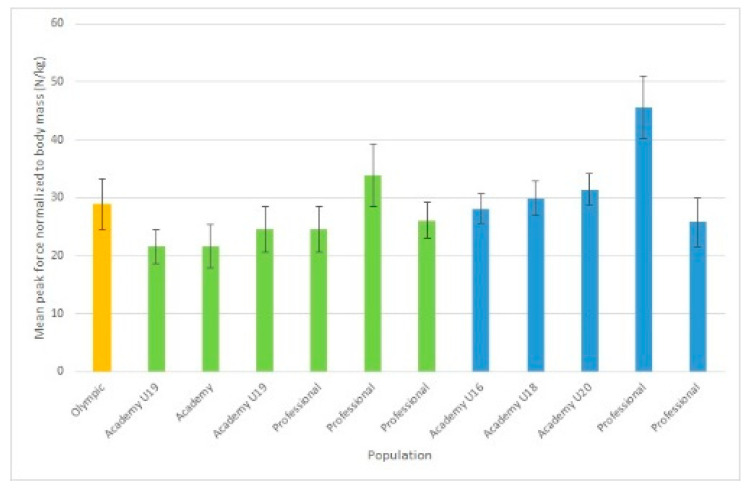
Reported Mean Total-Body Force Production Scaled to Player Mass. Notes: Orange = Rugby 7s; Green = Rugby League; Blue = Rugby Union.

**Figure 5:**
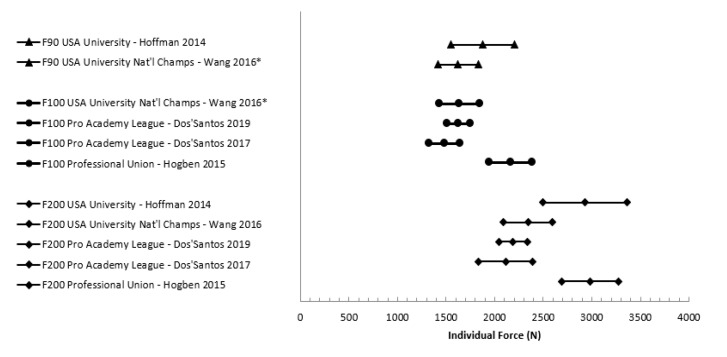
Average Force Development at 90, 100, and 200 ms. Notes: F90: force at 90 ms, F100: force at 100 ms, F200: force at 200 ms. * body mass not included in reported values.

**Figure 6:**
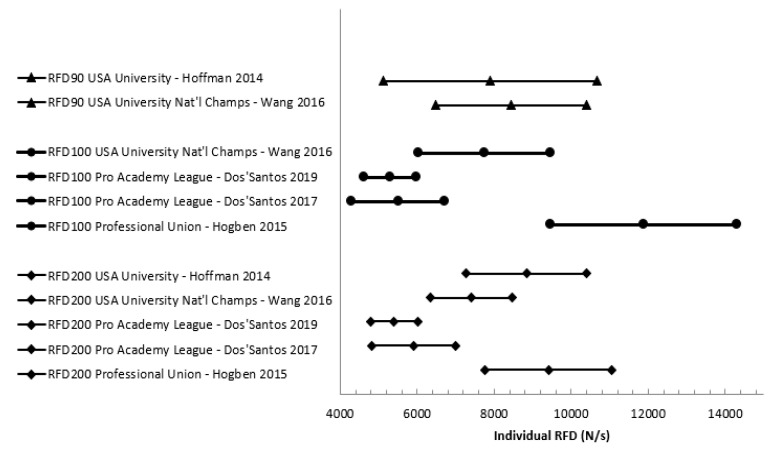
Average Rate of Force Development at 90, 100, and 200 ms. Notes: RFD90: rate of force development at 90 ms, RFD100: rate of force development at 100 ms, RFD200: rate of force development at 200 ms.

Twenty-three of the 24 studies reported peak force, but only eight reported rate of force development and only one reported impulse. One study [29] did not provide any of the force production variables of interest for data extraction; however, upon enquiry, the authors provided peak force and rate of force production means and standard deviations from that research. Additionally, upon enquiry, Dos’Santos et al. [30] (reference reflects amended manuscript) indicated that the results they originally reported needed to be amended, and they sent updated data for use in this review. Reported mean peak force from the IMTP can be seen in Figure 3. While most studies reported force in N, some reported it in kg. Note that for Figure 3, any peak force results reported in anything besides N were converted to make comparable data in the graph; for example, if the article reported peak force in kg, then it was multiplied by 9.81. Additionally, there was a lot of variance in reporting if peak force was inclusive of body weight or not, and it could not always be determined which was the case—ambiguous peak forces in the figure are reported without distinction of if they were gross or net force. Across studies, only 12 reports of force normalized for athletes’ body mass were given, and they are summarized in Figure 4. Means and 95% confidence intervals of reported instantaneous force and mean rate of force development at standard time points was summarized in forest plots (Figure 5 and Figure 6).

Rating scores from the QAT can be seen in Table 4. Major strengths of most studies included well-defined samples, high participation rates of players within a team, and citing a prior procedural use of the IMTP. None of the studies reported a sample size calculation, though in the context of research on a sports team this is usually not practical to do a priori, because potential sample size is low compared to clinical trials (for example, there may only be 25 players total on a team from which to draw a sample). However, only 52% of studies calculated effect size, which more studies should do to provide additional context to null hypothesis significance tests, which generally suffer from low sample size. None of the assessors were blinded to the exposure status of participants; again, this criteria may not be feasible to meet in studies of athletes, even using samples at different playing levels. Based on the QAT criteria, studies averaged a quality score of 73.2%, indicating moderate quality of reporting.

Rating scores from assessment of secondary outcomes about the IMTP procedures can be seen in Table 1. While the majority of studies used a force plate to measure results of the IMTP, four studies [24,26,33,41] exclusively used a dynamometer, which means that many of the criteria were not applicable, such as sampling rate of the force plate. Based on the criteria from Table 1, most studies (*n* = 17) rated below 50%, with the average quality rating for the studies being 40.6% (Table 1), indicating poor quality of reporting of IMTP procedures. Some of the important procedural criteria that were commonly not reported include if athletes underwent familiarization on the test and if participants were secured to the bar with wrist straps and/or athletic tape. Additionally, the strictness and detail of reporting of the knee and hip angles the athletes used during testing was quite variable. One of the most common statements was narrative in nature, stating that athletes got into a position representing the second pull of the power clean. Data capture and processing were the areas with the least detail reported, except for the sampling rate of the force plate. Few studies reported how they detected the onset of the pull, provided sufficient detail on if and how body weight was accounted for, or if any smoothing or filtering techniques were used in processing the data.

## 4. Discussion

The main goals of this review were to summarize any data collected using the IMTP test in any rugby players; to create normative data for this test for coaches and players to use; and to critique the methodology studies used and reported in conducting their research. While this review provides a summary of the available data on IMTP performance among rugby players across three different codes of rugby and many playing levels, the inconsistent reporting of results prohibited the formation of true normative data. However, this review makes a significant contribution by providing researchers with a comprehensive and detailed list of what items to control for during IMTP testing and to report in their manuscripts. While there was an overall trend for greater force production with an increase in playing level and age, there is a great deal of heterogeneity between the studies. For example, one study indicated that professional rugby league players [26] generated less force than what another study reported English adolescent rugby players could generate [43]. Strength and power should be able to discriminate between playing levels in rugby [46]. Within professional rugby league players, the population most frequently studied, there was over a 1900 N/200% difference in reported strength between the lowest [26] and highest means reported in different studies [36]. The wide variability in reported peak force found at different playing levels in this review could not conclusively show the expected discrimination, likely due to lack of standardization in procedures rather than any true similarity in peak force production between professional and U18 players. Individual studies that specifically compared different levels of play/age group within the same study [27,28,33,36] found that, in general, peak force production increased with age and playing level. As the testing methods, analytical methods, and modifications of the reported values (e.g., gross versus net force) are reported to be consistent within each of these four between-group comparison studies, we can conclude that greater age group and level of play are associated with greater force production [27,28,33,36]. We cannot, however, make this same conclusion based upon the sum of all of the literature included in the present study, due to the substantial variability in reporting and procedures among studies. Therefore, we do not feel able to meet our stated purpose of creating acceptable normative data, though the data presented here can form a preliminary basis for comparison until better quality data become publicly available.

Measures of early force production (i.e., force at specific time points and RFD) also suffer from a lack of standardization. Based on prior data suggesting increases in performance with greater levels of play, one would expect a general trend of increasing average RFD and instantaneous force at specific time points with level of play. This does not appear to be the case when comparing results across studies. The mean values displayed in Figure 4 and Figure 5 do not show clearly increasing performance with each level of play as would be expected (i.e., one would expect RFD100 for USA university national champion players to be lower than professional academy league players, which would likely also be lower than professional players). Issues with standardization such as inclusion or exclusion of body weight make comparison difficult. For example, all studies included in Table 4 include the force of body weight in the reported values except for Wang et al. [44]. Not all studies were clear about the inclusion or exclusion of body mass in the paper, and the corresponding authors were contacted [30,31,35,44]. These methodological issues lead to difficulty in clearly understanding the trends that may or may not exist between playing levels for these early force-time variables. Of the 24 studies included in this review, while all report peak force, only 5 studies reported instantaneous force or average rate of force development. The many variables calculated from the early force-time curve (e.g., impulse/RFD from the start of the pull to 200 ms, instantaneous force at 200 ms) provide a wealth of information relevant to rugby performance that should be included in future studies. Due to known influences of calculation methods for rate of force development on reliability and difficulty of comparison of different RFD constructs, we recommend future studies look to Haff et al. [21].

The results of this literature review indicate that, while the IMTP test for rugby players is gaining popularity in use among both researchers and coaches, there has been a lack of standardization on the procedure of conducting the IMTP test. This makes comparisons between studies difficult, as some of the differences in procedure, such as body positioning [47,48], reporting of gross (include body weight) versus net values (exclude body weight) [20], and onset detection method [31,49] have been shown to influence reported force output on the test. This lack of accepted standards and quality control has been noted in a wide variety of physiological measures used to assess rugby players [50].

The average score for general study design rating using the National Institutes of Health’s recommended system for observational studies (i.e., the QAT) indicated moderate-to-good quality on average. However, that score is somewhat artificially lowered due to the circumstances of the literature not necessarily matching with the original intent of the rating tool. First, the worst-rated study was a conference poster [42], so it did not have the scope to report the level of detail a journal article could. Second, some common practices in clinical trials, such as sample size calculations, are often not feasible in sports settings, especially among elite athletes where the population is small. However, to compensate, studies should make sure they are reporting effect sizes or other measures of statistical power of their comparisons.

More important than general study design are the specific procedures and standards employed when conducting the IMTP test. While there is some debate on precisely which specifications are ideal for certain parameters such as optimal filtering and pull identification parameters, it should be universally agreed upon that a better level of detail of reporting is needed if we wish to make meaningful comparisons between studies for the IMTP. While the rating tool contained in Table 3 was designed to evaluate the adequacy of IMTP procedure reporting, it may also serve as a guide for future research to ensure that sufficient detail is included for later comparison and replication. In addition, the reader is referred to a recent review providing specific recommendations on best practices for using the IMTP test [15]. Utilization of both of these resources will increase the usefulness of IMTP results reported in the literature, and provide practitioners with a more comprehensive and more easily interpreted summary of rugby players’ abilities. We suspect that the issue of standardization and reporting is true across IMTP research in other populations, making these recommendations apply more broadly than to just rugby research.

While this review was limited to reporting and summarizing force production measured by the IMTP, in reading the studies, we noticed several opportunities for future research. Few of the studies reviewed used results from the IMTP to categorize or discriminate athletes by playing level or position or correlated results from the IMTP with other outcomes, and in those that did, the results were equivocal. Some studies have not indicated a relationship between IMTP and other measured factors. One study showed that IMTP force had a positive linear association with injury risk among school-boy rugby players [33]; however, in their multivariate analysis of what contributed to risk injury, it appears that IMTP force is not a meaningful predictor of injury rates. Similarly, in university rugby 7s players, when IMTP force production was entered into a regression model with other physical characteristics, it did not meaningfully contribute to injury rates [40]. Surprisingly, Quarrie and Wilson [41] did not find a significant correlation between IMTP and individual scrum force among men competing in the Dunedin premier rugby competition. In contrast to these three examples, other studies have shown strong relationships between IMTP outcomes and other fitness and performance outcomes. For example, Wang et al. [44] found that RFD measured in IMTP significantly correlated with measures of strength, speed, and agility in collegiate rugby players. Additionally, Hoffman [34] found significant correlations between IMTP force and resisted sprinting speed. West and colleagues [45] found that, among professional rugby league players, unscaled peak force did not correlate with vertical jump power or sprinting speed, but force scaled to body weight did. These studies have mostly focused on correlations within different general fitness and performance outcomes. Future research should examine the relationship between outcomes from the IMTP and other measures of rugby-specific fitness and specific in-game actions like making, breaking, or evading tackles, rucking, and scrumming.

Strengths of the present review include inclusion of gray literature, face validation of the quality of IMTP reporting tool by multiple experts in this area, and follow-up with authors to clarify their results when needed. Additionally, having the review protocol registered a priori helps with the transparency of the review. Finally, having both authors independently execute both the search and screening protocols limits the bias introduced into the inclusion and data extraction of articles. While this study has significant strengths, there are limitations worth mentioning. First, the systematic search for publications included only studies written in English. Additionally, while the rating tool of IMTP test procedures reporting was developed with multiple rounds of feedback from IMTP experts and met face validity, no more stringent testing of the tool was undertaken at this time. Finally, the QAT tool used for rating general methodological quality may not have been specifically suited to the types of studies collected; however, as a well-recognized instrument, it was deemed the best-available for the scope of this review.

It is clear from the body of research that there are substantial gaps in the literature for certain populations. First, very little data exist for female rugby players. These players represent a significant proportion of the overall player base, yet there are few studies that have evaluated female players in any capacity [51], let alone using the IMTP. In addition, it is clear that the majority of data has been collected in Europe; few data have been collected in other areas of the world in which rugby is both popular and is played at a high level. While three studies were conducted in New Zealand, there is ample opportunity for research in other countries such as South Africa, Argentina, and Australia. We recommend that future research be done in these yet-unstudied populations to address these gaps.

Some practical recommendations can be made from the general trends found in the present systematic review, the findings of studies which directly compare different playing levels [27,28,33,36], and the findings of studies evaluating the relationship of force production characteristics with rugby skills [8,9,10,11,12,13]. First, greater levels of strength and rate of force development are important for sporting success and ascending to higher levels of competition. The training necessary to achieve these qualities (i.e., strength training) serves to prepare the athlete to compete well and be resilient to injury [52]. Second, the IMTP is a useful test of force-production-related fitness qualities that is both safe and efficient [53]. With greater IMTP research done with rugby populations, better normative data will emerge, providing useful points of comparison for rugby athletes.

## 5. Conclusions

In conclusion, the IMTP test is a popular tool for evaluating full-body force production and has significant strengths, making it a worthwhile tool for evaluating rugby players. However, standardization of methodology is a critical aspect of ensuring that data collected using this test are accurate, valid, and allow for comparison between studies. Across the literature, there is broad variation in the methods used and reported, making it difficult to make accurate conclusions about rugby players across different playing levels. Going forward, there is a great need for extensive standardization across studies to maximize the usefulness of this test and the data obtained from its use. Therefore, we strongly recommend that future coaches and researchers, within rugby or any other population, follow the procedures outlined by Comfort et al. [15] and, in order to improve reporting of IMTP methods, use the rating tool included in this paper as a guide for reporting study methods.

## Figures and Tables

**Table 1:** IMTP Test Procedure Reporting Rating Tool.

Item #	Outcome Assessed	Scoring Guide
1	Participant’s sport experience	0 = not reported 1 = reported generally only (e.g., experienced) 2 = reported specifically (e.g., had played for at least 1 full season)
2	Participant competitive level	0 = not reported Investigative 1 = reported in generally only (e.g., well-trained) 2 = reported specifically (e.g., Academy Rugby League players)
3	Participant’s resistance training experience	0 = not reported 1 = reported generally only (e.g., had some resistance training experience) 2 = reported specifically (e.g., >2 years of resistance training experience of >2 time per week)
4	Timing of assessment relative to competition calendar and training phase	0 = not reported 1 = reported generally only (e.g., during preseason) 2 = reported specifically (e.g., last week of preseason and end of taper)
5	Familiarization Procedure	0 = not reported 1 = reported generally only (e.g., familiarization was conducted 48 h prior) 2 = reported specifically (e.g., familiarization of 3 s each of 75% and 90% maximum effort trials completed 48 h prior to testing)
6	IMTP Specific Warm-Up Procedure on Day of Main Testing	0 = not reported 1 = reported generally only (e.g., athletes warmed up at a submaximal weight) 2 = reported specifically (e.g., athletes warmed up by performing 3 s each of 75% and 90% maximum effort trials)
7	Use of wrist straps and athletic tape	0 = not reported 1 = explicitly reported that only straps or straps and athletic tape were used
8	Knee angle	0 = not reported 1 = reported generally only (e.g., in the position representing the second pull of the power clean) 2 = reported estimated knee angle (e.g., 120–130 degrees) 3 = reported actual/measured knee angle (e.g., 125 ± 7 degrees)
9	Hip angle	0 = not reported 1 = reported generally only (e.g., in the position representing the second pull of the power clean) 2 = reported estimated hip angle (e.g., 130–140 degrees) 3 = reported actual/measured hip angle (e.g., 145 ± 7 degrees)
10	Reporting of Pull Onset Detection Method	0 = not reported 1 = reported generally (e.g., used autodetection of pull start) 2 = reported specifically (e.g., used autodetection of pull start using 10 N as threshold, used manual identification)
11	Reporting of Gross (includes force due to body weight) or Net (does not include force due to body weight) Force Values from the Force Plate	0 = not reported 1 = reported that values did or did not include body weight
12	Reporting of sampling rate	0 = not reported 1 = reported sampling rate
13	Data Processing	0 = did not report smoothing or filtering methods used to process data 1 = reported smoothing or filtering methods or lack thereof (e.g., 4th order Butterworth Low pass Filter at 10 Hz)
14	Rate of Force Development	0 = did not report method used for calculating rate of force development 1 = reported with enough detail to adequately identify which RFD measures was used (e.g., peak RFD with 5 ms sampling window, average RFD from 0–200 ms)

Notes: IMTP = isometric mid-thigh pull.; RFD = rate of force development.

**Table 2:** Scoring of Studies Using IMTP Test Procedure Reporting Rating Tool.

Item from IMTP Rating Tool	1	2	3	4	5	6	7	8	9	10	11	12	13	14	Paper Score Across Items
Atkins 2004 [24]	1	2	1	2	0	0	0	3	0	NA	NA	NA	NA	NA	9/19 = 47%
Bourgeois et al. 2017 [19]	0	0	2	1	0	1	0	3	3	0	0	1	0	0	11/25 = 44%
Crewther et al. 2012a [18]	1	2	1	0	0	0	1	1	1	0	0	1	0	0	8/25 = 32%
Crewther et al. 2012b [25]	1	2	1	0	0	0	1	1	1	0	0	1	0	0	8/25 = 32%
Crewther et al. 2016 [26]	2	2	0	2	1	1	0	1	1	NA	NA	NA	NA	NA	10/19 = 53%
Darrall-Jones et al. 2015 [27]	0	2	0	2	0	1	0	2	2	0	0	0	0	0	9/25 = 36%
Dobbin et al. 2018 [28]	2	2	2	1	0	2	0	2	0	0	0	1	0	0	12/25 = 48%
Dobbs et al. 2017 [29]	0	1	0	0	0	2	0	3	1	0	0	1	0	0	8/25 = 32%
Dos’Santos et al. 2019 [30]	1	2	2	2	0	2	1	1	1	2	0	1	1	1	17/25 = 68%
Dos’Santos, Jones, et al. 2017 [31]	1	2	2	2	0	2	0	0	0	2	0	1	0	1	13/25 = 52%
Dos’Santos et al. 2017 [32]	1	2	2	1	0	2	1	1	1	2	0	1	0	1	15/25 = 60%
Hislop 2017 [33]	0	0	0	1	0	2	0	1	1	NA	NA	NA	NA	NA	5/19 = 26%
Hoffmann 2014 [34]	2	2	0	0	0	2	1	3	3	0	0	1	0	1	15/25 = 60%
Hogben 2015 [35]	2	2	2	2	0	0	1	2	0	0	0	0	0	1	12/25 = 48%
Ireton et al. 2017 [36]	1	2	0	1	0	1	1	2	2	0	0	0	0	0	10/25 = 40%
La Monica et al. 2016 [37]	0	2	0	1	0	0	0	1	1	2	0	1	0	1	9/25 = 36%
Marrier et al. 2017 [38]	1	2	0	2	0	0	0	3	3	0	0	1	0	0	12/25 = 48%
McMaster et al. 2017 [39]	1	1	0	0	0	1	0	0	0	0	0	0	0	0	3/25 = 12%
Mirsafaei Rizi et al. 2017 [40]	2	2	0	1	0	0	0	0	0	0	0	0	0	0	5/25 = 20%
Quarrie and Wilson 2000 [41]	0	2	0	0	0	1	0	2	2	NA	NA	NA	NA	NA	7/19 = 37%
Tavares and Mil-Homens 2015 [42]	1	1	0	0	0	0	0	0	0	0	0	0	0	0	2/25 = 8%
Till et al. 2018 [43]	0	0	0	1	1	1	0	1	1	0	1	1	0	0	7/25 = 28%
Wang et al. 2016 [44]	2	2	0	0	0	0	0	1	1	0	1	1	0	1	9/25 = 36%
West et al. 2011 [45]	1	2	2	2	1	0	1	2	2	2	1	1	1	0	18/25 = 72%
**Item average across studies**	**1.0**	**1.6**	**0.7**	**1.0**	**0.1**	**0.9**	**0.3**	**1.5**	**1.1**	**0.5**	**0.2**	**0.7**	**0.1**	**0.4**	**Average Paper Score across studies = 40.6%**

Notes: NA = not applicable.

**Table 3:** Summary of Included Studies.

Study	Population	Time of Season	Knee and Hip Angles	PF Measured	RFD Measured	Impulse Measured
Atkins, 2004 [24]	18 first team (22.3 ± 4.6 years), 16 alliance (18.8 ± 0.8 years), and 20 academy (17.2 ± 0.9 years) players from an elite English rugby league franchise	First week of preseason	Knee angle of 135° measured by goniometer; hip angle not reported	Yes	No	No
Bourgeois et al. 2017 [19]	16 high-school-aged rugby union players in New Zealand (15.0 ± 0.9 years, 80.2 ± 15.3 kg) with at least 6 months of organized resistance training experience	off-season	Knee angle: 125°; Hip angle: 140°	Yes	No	No
Crewther et al. 2012a [18]	79 professional rugby union players in England (103.2 ± 12.4 kg, did not report mean age but gave range of 18–32 years)	Not specified	Not reported	Yes	Yes	No
Crewther et al. 2012b [25]	64 professional rugby union players in England	Not specified	Knee angle of 120–130°; did not report hip angle	Yes	No	No
Crewther et al. 2016 [26]	12 professional male rugby league players in Australia (23.4 ± 3.6 years old, 95.4 ± 11.0 kg)	during the competitive season; testing session took place 2–4 days after a game	Not reported	Yes	No	No
Darrall-Jones et al. 2015 [27]	67 male English regional academy rugby union under 16 s, *n* = 29; under 18 s, *n* = 23; under 21 s, *n* = 15	Beginning of preseason; Testing took place after a 6-week off-season training period whereby all players completed a 3-week preparation program including full-body resistance training, aerobic conditioning running, and speed technique sessions.	Knee angle: ~120–130° (didn’t measure, just cited instructions of prior research); hip angle: did not report	Yes	No	No
Dobbin et al. 2018 [28]	male rugby league players with at least 2 years’ resistance training experience, 33 classified as senior players who had completed at least one season of competition in the English Super League (25.3 ± 3.4 years, 97.9 ± 9.5 kg) and 23 currently at Academy level or who had graduated to the first team in the last 3 months (18.3 ± 1.4 years, 86.2 ± 8.2 kg)	preseason	Knee angle: ~140°; did not report hip angle	Did not report; received in personal communication	Did not report; received in personal communication	No
Dobbs et al. 2017 [29]	American amateur rugby union players, 11 females (24.73 ± 3.66 years old, 74.00 ± 18.14 kg) and 6 males (22.0 ± 2.61 years old, 80.28 ± 11.13 kg)	Prior to the competitive season	Knee angle: 135°; did not report hip angle	Yes	Yes	No
Dos’Santos et al. 2019 [30]	30 professional academy rugby league players in England (17.5 ± 1.1 years, 85.4 ± 10.3 kg) with at least 2 years of weight training experience	2nd week of preseason mesocycle	Self-selected	Yes	Yes	No
Dos’Santos, Jones, et al. 2017 [31]	9 professional academy rugby league players in England (18.5 ± 0.4 years, 91.2 ± 13.1 kg) with at least 2 years of weight training experience	2nd week of preseason mesocycle	Self-selected	Yes	Yes	No
Dos’Santos et al. 2017 [32]	35 male professional rugby league players in England (24.2 ± 4.8 years old, 94.5 ± 11.5 kg)	End of preseason	Self-selected	Yes	No	Yes
Hislop 2017 [33]	A total of 399 school boy rugby union players in England (*n* = 138 U15s, 67.1 ± 11.8 kg; *n* = 92 U16s, 73.6 ± 14.4 kg; and *n* = 169 U18s, 78.6 ± 10.3 kg)	preseason	Not reported	Yes	Yes	No
Hoffmann 2014 [34]	11 male American university club rugby union players, played at least 1 year Mean age 21.9 ± 2.5 years Mass 80.6 ± 12.5 kg	Not reported	Knee: 125 ± 5°; Hip: 175 ± 5°	Yes	Yes	No
Hogben 2015 [35]	19 professional English rugby union players on team for at least one year and with at least 2.5 years’ resistance training experience (age 26.0 ± 5.1 years, 105.1 ± 14.8 kg)	start of preseason	Knee angle of 140°; did not report hip angle	Yes	No	No
Ireton et al. 2017 [36]	18 Senior (25.5 ± 4.5 years, 97.1 ± 12.6 kg), 23 U19 (17.7 ± 0.9 years, 87.0 ± 8.8 kg), and 14 U16 (15.3 ± 0.5 years, 78.3 ± 12.4 kg) academy rugby league players in England	Beginning of preseason period	Knee angle ~120–130°, did not report hip angle	Yes	Yes	No
La Monica et al. 2016 [37]	25 male American collegiate rugby players (20.2 ± 1.6 years, 82.4 ± 13.2 kg)	preseason	Self-selected	Yes	No	No
Marrier et al. 2017 [38]	10 male French Olympic rugby 7s players (26 ± 5 years, 90 ± 11 kg)	preseason	Knee angle of 140 ± 7°; Hip angle of 138 ± 13°	Yes	No	No
McMaster et al. 2017 [39]	10 well-trained male rugby players in New Zealand (21.0 ± 2.6 years, 95.7 ± 10.8 kg)	Not reported	Not reported	Yes	No	No
Mirsafaei Rizi et al. 2017 [40]	90 male (20.73 ± 2.06 years, 70.8 ± 9.56 kg) and 14 female (20.30 ± 1.16 years, 53.3 ± 5.10 kg) university rugby 7s players, with an average playing experience of 21.0 months (range 0–144 months; 28 players were completely new to the sport)	preseason	Not reported	Yes	No	No
Quarrie and Wilson 2000 [41]	56 male rugby forwards in the Dunedin premier competition (23.2 ± 3.1 years, 183 ± 8 cm, 96.9 ± 9.8 kg)	Not reported	Knee angle was set between 115° and 125° (angles were checked with a large protractor)	Yes	No	No
Tavares and Mil-Homens 2015 [42]	20 amateur male rugby players in Portugal (24.9 ± 5.12 years, 89.2 ± 11.8 kg)	Not reported	Not reported	Yes	No	No
Till et al. 2018 [28]	22 male adolescent rugby league players in England (15.3 ± 0.5 years, 77.0 ± 13.3 kg)	preseason	Not reported	Yes	Yes	No
Wang et al. 2016 [44]	15 male American rugby union players from university club rugby defending National Champions team (20.67 ± 1.23 years, 86.51 ± 14.18 kg), with 1–6 years’ playing experience	Not reported	Self-selected	Yes	No	No
West et al., 2011 [45]	39 professional rugby league players in England (24.0 ± 4.6 years, 97.0 ± 8.2 kg) with at least 2 years’ resistance training experience	Between preseason and start of competitive season	Knee angle of 120–130°; hip angle not reported	Yes	Yes	No

Note: PF = Peak Force; RFD = Rate of Force Development.

**Table 4:** Quality Assessment Tool for Observational Cohort and Cross-Sectional Studies Ratings of Included Studies.

Study	Q1	Q2	Q4	Q5	Q8	Q9	Q11	Q12	Q14	Total Score
Atkins 2004 [24]	Y	Y	Y	N	Y	Y	Y	N	NA	6/8 = 75%
Bourgeois et al. 2017 [19]	Y	Y	Y	N	Y	Y	Y	N	N	6/9 = 67%
Crewther et al. 2012a ^a^ [18]	Y	Y	Y	N	Y	Y	Y	N	Y	6/9 = 67%
Crewther et al. 2012b ^a^ [25]	Y	Y	Y	N	NA	NA	Y	N	Y	5/7 = 71%
Crewther et al. 2016 [26]	Y	Y	Y	N	NA	NA	Y	N	N	4/7 = 57%
Darrall-Jones et al. 2015 [27]	Y	Y	Y	N	Y	Y	Y	N	Y	7/9 = 78%
Dobbin et al. 2018 [28]	Y	Y	Y	Y	Y	Y	Y	N	Y	8/9 = 89%
Dobbs et al. 2017 [29]	Y	N	N	N	Y	Y	N	N	Y	4/9 = 44%
Dos’Santos et al. 2019 ^b^ [30]	Y	Y	Y	Y	Y	Y	Y	N	Y	8/9 = 89%
DosʼSantos, Jones et al. 2017 ^b^ [31]	Y	Y	Y	Y	Y	Y	Y	N	Y	8/9 = 89%
Dos’Santos et al. 2017 [32]	Y	Y	N	Y	Y	Y	Y	N	Y	7/9 = 78%
Hislop 2017 ^c^ [33]	Y	Y	Y	Y	Y	Y	N	N	Y	7/9 = 78%
Hoffmann 2014 ^c^ [34]	Y	Y	Y	Y	NA	NA	Y	N	Y	6/7 = 86%
Hogben 2015 ^c^ [35]	Y	Y	Y	Y	NA	NA	Y	N	Y	6/7 = 86%
Ireton et al. 2017 [36]	Y	Y	Y	Y	Y	Y	Y	N	N	7/9 = 78%
La Monica et al. 2016 [37]	Y	Y	Y	Y	Y	Y	Y	N	N	7/9 = 78%
Marrier et al. 2017 [38]	Y	Y	Y	Y	Y	Y	Y	N	Y	8/9 = 89%
McMaster et al. 2017 [39]	Y	N	N	Y	Y	Y	N	N	NA	4/8 = 50%
Mirsafaei Rizi et al. 2017 [40]	Y	Y	Y	N	Y	Y	N	N	N	5/9 = 56%
Quarrie and Wilson 2000 [41]	Y	Y	Y	Y	NA	NA	Y	N	Y	6/7 = 86%
Tavares and Mil-Homens 2015 [42] ^d^	Y	Y	Y	N	NA	NA	Y	N	NA	4/6 = 67%
Till et al. 2018 [28]	Y	N	Y	Y	NA	NA	Y	N	Y	5/7 = 71%
Wang et al. 2016 [44]	Y	Y	Y	N	NA	NA	Y	N	N	4/7 = 57%
West et al. 2011 [45]	Y	Y	Y	N	NA	NA	Y	N	Y	5/7 = 71%
*n* (%) that met criteria	24/24 (100%)	21/24 (87.5%)	21/24 (87.5%)	13/24 (54.2%)	15/15 (100%)	15/15 (100%)	20/24 (83.3%)	0	15/24 (62.5%)	Average score = 73.2%

Notes: ^a^: these two papers come from the same study; ^b^ these two papers come from the same study; ^c^: thesis or dissertation; ^d^: conference abstract. NA = not applicable.
